# Preparation of Biochar from Papermaking Sludge and Its Adsorption Characteristics for Tetracycline

**DOI:** 10.3390/toxics13121050

**Published:** 2025-12-04

**Authors:** Jiayu Niu, Siyuan Fan, Zhenjun Wu

**Affiliations:** 1 School of Environmental Engineering, Henan University of Technology, Zhengzhou 450001, China; niujiayu@haut.edu.cn (J.N.);; 2 Zhengzhou International Cooperation Base for Science and Technology on Carbon Neutrality of Organic Solid Waste Conversion, Zhengzhou 450001, China; 3 Henan International Joint Laboratory of Environmental Pollution Remediation and Grain Quality Security, Zhengzhou 450001, China

**Keywords:** biochar, adsorption, papermaking sludge, tetracycline

## Abstract

Papermaking sludge, rich in intrinsic resource value, is effectively barred from direct deployment in environmental remediation, agriculture, or energy generation by its pronounced contaminant burden. Pyrolytic conversion into high-value paper sludge biochar, such as papermaking sludge biochar (PSBC) provides a green, efficient portal for closing its resource loop. In this study, papermaking sludge was converted into a series of paper sludge biochars (PSBCs) via oxygen-limited pyrolysis at 500–900 °C. The porous architecture, surface physicochemical properties, and crystalline structure of the biochars were comprehensively characterized, and their performance for aqueous tetracycline (TC) removal was systematically quantified. Pyrolysis at 900 °C afforded PSBC 900 with the lowest yield (36.05%) yet the highest Brunauer–Emmett–Teller (BET) surface area (79.53 m^2^/g), an extensively developed mesopore network, and the greatest degree of graphitization. Across an initial tetracycline (TC) concentration window of 20–160 mg/L, PSBC 900 delivered an equilibrium capacity (q_e_) of 72.22 mg/g, outperforming PSBC 700 and PSBC 500 by factors of 1.3 and 1.8, respectively. Optimal uptake was achieved at a dosage of 1.0 g/L, pH 7, and 120 min contact time. Among the background cations examined, Mg^2+^ exerted a pronounced inhibitory effect, whereas Na^+^, K^+^, and Ca^2+^ exerted negligible interference. The adsorption process was accurately described by the pseudo-second-order kinetic model and the Langmuir isotherm (R^2^ > 0.999), yielding a theoretical maximum capacity (q_m_) of 76.39 mg/g for PSBC 900 at 313 K. Thermodynamic parameters ($∆G^{\theta }$ < 0, $∆H^{\theta }$ > 0, $∆S^{\theta }$ > 0) confirm a spontaneous, endothermic, and entropy-driven process. After five consecutive adsorption–desorption cycles, PSBC 900 retained >64.68% of its original efficiency, demonstrating excellent regenerability. Paper sludge biochar enables a “waste-to-treat-waste” strategy for the efficient abatement of tetracycline, offering an economically viable and high-performance technology that advances the remediation of tetracycline-laden wastewaters.

## 1. Introduction

Industrial wastewater created during production and the by-product of paper sludge produced during wastewater treatment procedures are the main sources of environmental pollution associated with the paper industry [1]. According to reports, the output of paper sludge is around five to ten times that of papermaking sludge, and it ranks first across all industries in terms of sludge production [2]. Papermaking sludge’s complex composition, which includes cellulose, natural or synthetic binders, inks and dyes, nutrients like phosphate and nitrogen, and biological and bacterial metabolic wastes, makes sludge dewatering and reprocessing considerably more challenging. The great majority of the organic matter in paper sludge decomposes anaerobically, and it is prone to rotting and releasing an unpleasant odor, which degrades the quality of the surrounding environment. Additionally, the sludge’s toxic and harmful bacteria and viruses cause diseases to spread over a wide area, posing a serious threat to human health. Nowadays, paper firms typically use conventional treatment techniques including burning, open piles, or landfilling. They all result in varying levels of secondary pollution and even severe environmental contamination, which creates several adverse issues for the growth of the paper sector and the advancement of ecological health. Paper sludge that is high in biomass and organic matter is a useful material for making biochar, according to an analysis of its nature [3].

Tetracycline is widely used in the medical and aquaculture fields [4]. Currently, to address the challenge of tetracycline remediation in wastewater, various technologies for removing tetracycline from water have been developed, including biological methods, membrane separation, coagulation, photolysis, and chemical oxidation [5]. However, these methods have various limitations, such as high material costs, high energy consumption, and secondary pollution caused by the additional use of chemical reagents. Adsorption, on the other hand, has become the primary method for removing antibiotic pollutants such as tetracycline in practical applications due to its advantages of high efficiency, low cost, simple process, low energy consumption, and no secondary pollution [6]. Biochar is a type of novel carbonized material that is currently widely used. It denotes a carbon-rich solid product generated via the anaerobic pyrolysis of biomass at elevated temperatures for a defined residence time, characterized by a stable porous framework, abundant surface functional groups, high specific surface area, and elevated carbon content. Biochar feedstocks are inexpensive and readily accessible; typical precursors include crop residues, poultry manure, nut shells, lignocellulosic biomass, municipal solid waste, and other organic waste [7]. The adsorption capacity of biochar is ultimately governed by its key physicochemical attributes—namely pH, surface charge, functional-group density, specific surface area, and pore architecture [8]. Owing to these favorable characteristics, biochar has been widely deployed as a low-cost, high-efficiency adsorbent for the removal of inorganic/organic contaminants and heavy-metal ions from aqueous matrices. Wang produced rice-straw biochar by anaerobic pyrolysis at 400 °C and 600 °C and subsequently evaluated its performance for tetracycline (TC) sequestration from synthetic wastewater. Biochar synthesized at 600 °C exhibited markedly higher TC uptake within 20 h. A parallel assay using swine manure biochar generated under identical conditions reached equilibrium after 36 h and likewise displayed superior removal at 600 °C; nevertheless, rice-straw biochar delivered the overall higher adsorption efficiency, underscoring the precursor-dependent nature of biochar reactivity [9]. These unique physical and chemical properties, combined with the wide range of sources of its preparatory raw materials, have made biochar increasingly useful as a green, economical, efficient, and environmentally friendly adsorbent.

Tetracycline, a relatively common organic pollutant in the field of wastewater treatment today, was the target pollutant in this study, which examined the effects of a number of variables on the removal of tetracycline by adsorption on the sludge’s biochar. These factors included the biomass feedstock, the dosage of biochar, the temperature of the pyrolysis of preparation, the initial mass concentration of the tetracycline solution, the concentration of various salt ions, and the pH of the reaction system. This study’s objective is to offer theoretical references for the creative application of paper sludge and the assessment of its impact on the elimination of tetracycline contaminants from aquatic environments.

## 2. Materials and Methods

### 2.1. Chemical Reagents

Tetracycline (C_22_H_24_N_2_O_8_) was obtained from Shanghai Maclean’s Biochemical Technology Co., Ltd., Shanghai, China). NaOH, HCl, NaCl, KCl, CaCl_2_, and MgCl_2_ were obtained from Kermel Chemical Reagent Co., Ltd., Tianjin, China). All chemicals were used as received, without additional purification.

### 2.2. Adsorbent Synthesis

The papermaking sludge utilized in this study was sourced from the wastewater treatment unit of a papermaking facility located in Zhengzhou, Henan Province, China. The raw sludge was initially subjected to a pretreatment process, which involved natural air drying in a cool, light-shielded environment, followed by drying in a constant-temperature oven at 105 °C for 24 h until a constant weight was achieved. The resulting dried sludge cakes were then mechanically crushed and sieved through a 200-mesh screen. The sieved sludge was stored in glass beakers, sealed with a layer of biodegradable film, and kept in a dry, dark environment for subsequent use.

Sludge biochar was prepared by high-temperature slow pyrolysis. Dried, ground papermaking sludge was weighed into covered quartz crucibles, placed in a tube furnace (MXG, Shanghai Weixing Furnace Industry Co., Ltd., Shanghai, China) and purged with N_2_. The furnace was heated at 5 °C min^−1^ to 500, 700, or 900 °C and maintained for 120 min under oxygen-limited conditions. The resulting biochar was crushed, sieved through a 100-mesh screen, rinsed with deionized water, dried at 105 °C to constant weight, and stored in brown bottles labeled PSBC 500, PSBC 700, and PSBC 900.

### 2.3. Reagents and Standard Solutions

An accurately weighed 1.000 g of tetracycline (TC) powder was dissolved in an appropriate volume of anhydrous methanol under magnetic stirring. The solution was subsequently transferred into a 1000 mL brown volumetric flask and diluted to the mark with methanol to obtain a stock solution with a concentration of 1000 mg/L. The stock solution was stored in a freezer at sub-zero temperatures to prevent degradation.

### 2.4. Preparation of Calibration Standards and Simulated Wastewater

A series of TC standard solutions were prepared by sequential dilution of the stock solution with deionized water to yield concentrations of 5, 10, 15, 20, 25, and 30 mg/L, simulating tetracycline-containing wastewater. Calibration standards were prepared volumetrically using Class A glassware to ensure accuracy.

UV–Vis spectrophotometry was conducted using a 722S spectrophotometer (Shanghai Precision & Scientific Instrument Co., Ltd., Shanghai, China). A full-wavelength scan of the TC standard solution revealed a maximum absorption peak at 360 nm. Deionized water was employed as the blank reference. The absorbance of each standard solution was measured at 360 nm in 1 cm quartz cuvettes. A calibration curve was constructed by plotting absorbance (Y-axis) against concentration (X-axis). Linear regression analysis yielded the equation: y = 0.0338x + 0.0027 with a coefficient of determination (R^2^) of 0.9997, indicating excellent linearity within the tested concentration range.

### 2.5. Experimental Set up and Adsorption Procedure

Batch adsorption experiments were conducted in 50 mL Erlenmeyer flasks. A tetracycline (TC) solution with an initial concentration of 20 mg/L was prepared by appropriate dilution of the stock solution. A total volume of 50 mL was transferred into each flask without adjusting the initial pH. A predetermined mass of biochar was then added to each flask, which was subsequently placed in a thermostatic orbital shaker (120 rpm) at room temperature (25 ± 1 °C) for 120 min.

After the adsorption period, the suspensions were withdrawn using a 10 mL syringe and filtered through a 0.45 μm hydrophilic membrane filter into clean 10 mL test tubes to remove solid particles. The residual TC concentration in the filtrate was determined using a UV–Vis spectrophotometer (Model 722S, Shanghai Precision & Scientific Instrument Co., Ltd., Shanghai, China) at the maximum absorption wavelength of 360 nm.

### 2.6. Adsorption Experiment

#### 2.6.1. Impact of PSBC Dosage on Tetracycline Removal Efficiency

To quantify the dose–response relationship, PSBC 500, PSBC 700, and PSBC 900 were tested at nine incremental levels: 0.1, 0.2, 0.3, 0.5, 0.7, 1.0, 1.2, 1.5, and 2.0 g/L. The initial pH of the 20 mg/L TC solution was used as measured (no adjustment). All other variables were held constant: 120 rpm agitation, 120 min contact time, and 25 ± 1 °C.

#### 2.6.2. Influence of Initial Tetracycline Concentration on Adsorption onto PSBC

The dependence of tetracycline (TC) uptake on initial concentration was examined over the range 20–160 mg/L for PSBC 500, PSBC 700, and PSBC 900. The native pH of each solution was employed without adjustment; all other variables remained fixed at 1.0 g/L PSBC dosage, 120 rpm agitation, 120 min contact time, and 25 ± 1 °C.

#### 2.6.3. Kinetics of Tetracycline Adsorption onto PSBC

The influence of contact time (5–120 min) on TC sequestration was evaluated for PSBC 500, PSBC 700, and PSBC 900 under the previously identified optimum dosage of 1.0 g/L. The initial pH was left unadjusted; temperature (25 ± 1 °C), agitation speed (120 rpm), and initial TC concentration (20 mg/L) were held constant throughout.

#### 2.6.4. Impact of Initial pH and Ionic Strength on TC Adsorption

The pH-dependent uptake of tetracycline (TC) was examined between pH 3.0 and 11.0 for PSBC 500, PSBC 700, and PSBC 900. Solution pH was adjusted with 1 mol/L NaOH or HCl; all other variables remained fixed at 25 ± 1 °C, 120 rpm/min, and 20 mg/L initial TC.

Ionic strength effects were subsequently probed by introducing NaCl, KCl, CaCl_2_, or MgCl_2_ at 0.0, 0.1, 0.5, and 1.0 mol/L while maintaining identical temperature, agitation, TC concentration, and contact time.

#### 2.6.5. Regeneration of PSBC

PSBC reusability was tested by repyrolysis—the same route used for its synthesis and previously validated for TC-loaded carbons. After equilibrium, the supernatant was discarded, the was solid dried (105 °C, 24 h), and then repyrolysed (300 °C, 1 h, and N_2_). The cooled char was reused under baseline conditions (1.0 g/L, 20 mg/L TC, 120 rpm/min, 25 °C, and unadjusted pH).

### 2.7. Data Analysis

The removal efficiency of TC, expressed as R (%), was calculated using the following equation, Equation (1):

(1) $$\mathrm{Residual}\;R\;(\%)=\frac{C_{0}-C_{t}}{C_{0}}\times 100$$

The adsorption capacity at time *t*, $q_{t}$ (mg/g), was determined as Equation (2):

(2) $$q_{t}=\frac{\left(C_{0}-C_{t}\right)\times V}{m}$$

where

*C*_0_ is the initial TC concentration (mg/L); 

*C_t_* is the residual TC concentration at time *t*;

*V* is the volume of the TC solution (L);

*m* is the mass of biochar used (g).

#### Adsorption Isotherms

Equilibrium adsorption isotherms were constructed at 20, 30, and 40 °C using TC concentrations spanning 20–160 mg/L. PSBC was dosed at 1.0 g/L and the suspensions were agitated at 120 rpm for 120 min. The amount of TC adsorbed at equilibrium (mg/g), was calculated with Equation (3).

(3) $$C_{eq}=\frac{\left(C_{0}-C_{t}\right)\times V}{m}$$

where

$C_{eq}$ is the equilibrium concentration of TC in solution;

*C*_0_ is the initial TC concentration (mg/L);

*C_t_* is the TC concentration at adsorption time *t*;

*V* is volume of the TC solution (L);

*m* is the mass of biochar used (g).

## 3. Results and Discussion

### 3.1. Effect of Pyrolysis Temperature on Biochar Yield

The performance of the adsorbents depends on the physical and chemical characteristics of the biochar [10]. In addition to the distinctive influence of the raw material source, the pyrolysis temperature at the time of preparation plays an equally important role, and the preparation of biochar at varying pyrolysis temperatures causes notable changes in its physical and chemical properties [11]. This study employs thermogravimetric analysis (TGA) to investigate the pyrolysis properties of papermaking sludge’s raw materials. When the raw materials are heated and decomposed, mass changes occur, and TG (thermogravimetric) and DTG (derivative thermogravimetric) analyses can reflect the thermal stability and pyrolysis behavior of the raw materials. Figure S1 shows the TG and DTG curves of the raw materials in the temperature range of 30~1000 °C.

**Figure 1:**
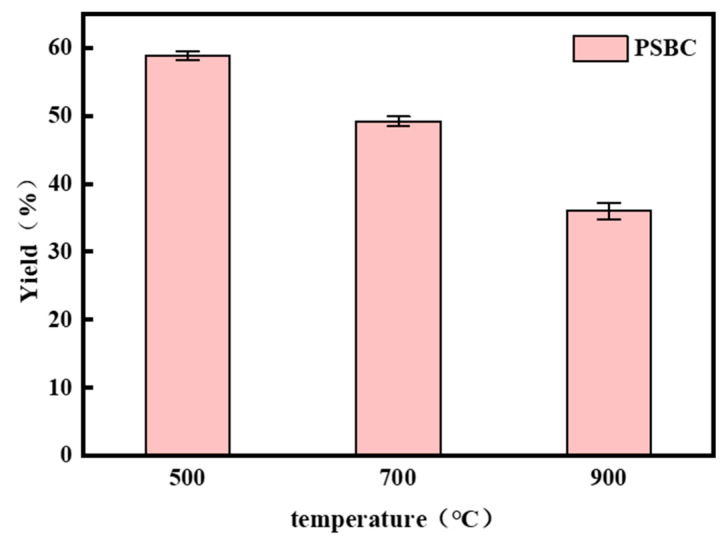
Biochar yield from papermaking sludge at pyrolysis temperatures of 500, 700, and 900 °C.

In order to prepare papermaking sludge biochar (PSBC 500, PSBC 700, and PSBC 900), high-temperature and slow pyrolysis at 500, 700, and 900 °C were chosen for this study based on the thermogravimetric analysis of raw sludge and related studies in the literature. This was performed in order to investigate the physicochemical properties of paper sludge biochar at various pyrolysis temperatures. As seen in Figure 1, the mass change before and after weighing was used to determine the yield of paper sludge at various temperatures.

Paper sludge biochar yields at 500, 700, and 900 °C were 58.91%, 49.24%, and 36.05%, respectively, as illustrated in Figure 1. The biochar yield dropped by 22.86% as the temperature increased from 500 °C to 900 °C. The pyrolysis temperature had a negative correlation with the total yield. In the papermaking sludge, the majority of the cellulose pyrolytically volatilizes in the form of volatile compounds, leaving behind an amorphous carbon matrix. At the same time, decarboxylation, decarbonylation, and dewatering reactions, among other processes, occur. This is because the high temperature encourages the full precipitation of bound water in the sludge and the continuous breakdown of the organic components into small organic molecules, which gradually form a porous biochar structure.

### 3.2. Characterization Analysis

#### 3.2.1. Analysis of Scanning Electron Microscope (SEM) Results

**Figure 2:**
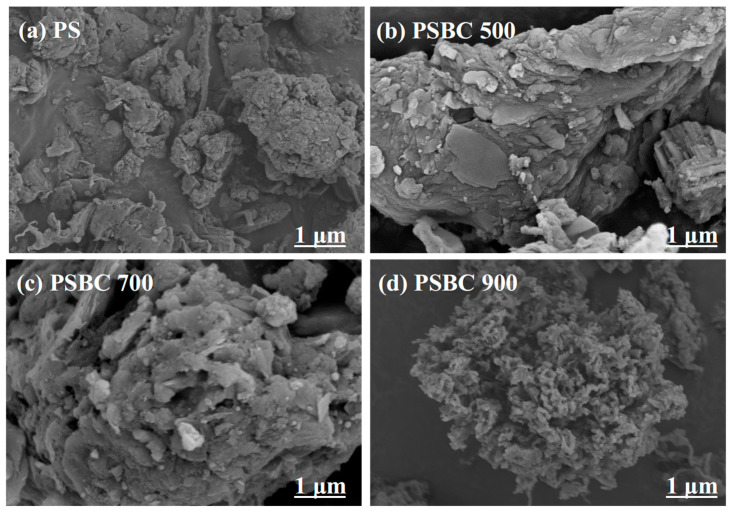
SEM diagram for papermaking sludge biochars (PS, PSBC 500, PSBC 700, and PSBC 900).

Figure 2a–d shows the SEM images of PS, PSBC 500, PSBC 700, and PSBC 900, respectively. The dried sludge without pyrolysis (PS) treatment is depicted in Figure 2a; the surface exhibits an uneven lumpy texture of varying sizes and nearly no discernible pore structure. The surface exhibited a lamellar structure and a little amount of pore structure resembling the dried sludge, PSBC 500, after pyrolysis treatment at 500 °C. The surface of PSBC 700 becomes rougher and more uneven than that of PSBC 500 as the pyrolysis temperature increases. The surface of PSBC 900 becomes even rougher and more porous, exhibiting a chaotic and intersecting mesh structure. This occurs because the temperature rise causes the water in the paper sludge to completely evaporate and cellulose and other small organic matter molecules to fully escape. Ultimately, the biochar’s skeleton is made up of only the remaining, hard-to-volatilize inorganic substances and carbon elements, which helps to form a porous structure and a larger specific surface area, which increases the biochar’s adsorption sites and fortifies its adsorption capacity [12].

#### 3.2.2. Analysis of Elemental Composition Results

Table 1 below displays the elemental analysis of the paper sludge biochars (PSBC 500, PSBC 700, and PSBC 900). The table shows that the elemental composition of paper-mill sludge biochar is significantly influenced by the pyrolysis temperature. As the pyrolysis temperature rises, there is a downward tendency in the amounts of the fundamental elements C, H, N, and O, as well as the H/C atomic ratio. On the other hand, there is a positive association between the (O+N)/C and O/C atomic ratios. As the pyrolysis temperature rose from 500 °C to 900 °C, the papermaking sludge biochar’s H/C ratio dropped from 0.05 to 0.02, indicating an extended aromatic framework, while O/C and (N+O)/C increased from 0.93 to 1.18 and 0.95 to 1.19, respectively. The concurrent rise in O/C and (N+O)/C reflects a higher relative abundance of O-/N-containing functional groups, the enhanced surface polarity lowers the solid–liquid interfacial energy, facilitating pollutant access to microporous active sites. The resulting aromatic–polar synergy boosts hydrophilicity and π–π electron–donor interactions, collectively upgrading the biochar’s physicochemical properties and tetracycline adsorption capacity. This suggests that when the temperature of pyrolysis rises, the paper-mill sludge biochar’s polarity increases, its hydrophilicity and aromaticity improve, and its physicochemical qualities improve [13].

#### 3.2.3. Analysis of X-Ray Diffraction (XRD) Results

**Figure 3:**
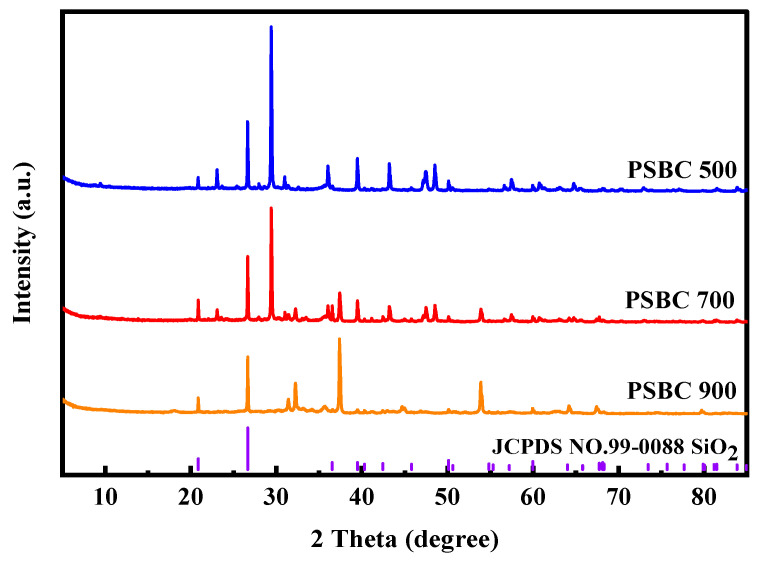
X-ray diffractograms diagram for PSBC 500, PSBC 700, and PSBC 900 obtained at different pyrolysis temperatures.

The X-ray diffractograms of the paper sludge biochars (PSBC 500, PSBC 700, and PSBC 900) are shown in Figure 3 below.

Peak morphology, as determined by XRD, is utilized to identify the crystal state of biochar; amorphous biochar is indicated by bun peaks, while crystalline biochar is indicated by sharp peaks [14]. A crystalline component of paper sludge biochar at varying temperatures is indicated by the existence of distinct, sharp diffraction peaks in multiple locations, displaying a typical graphitized structure. Additionally, as the pyrolysis temperature increased, several of the peaks diminished or vanished because the high temperature destroyed their crystal structure. Comparing biochar at various temperatures to the reference card (JCPDS NO.99-0088 SiO_2_), SiO_2_ components are present.

X-ray diffraction (XRD) patterns of paper-mill sludge biochars (PSBC 500, PSBC 700, and PSBC 900) are shown in Figure 3. Sharp, well-defined reflections superimposed on broad humps indicate a hybrid structure in which short-range-ordered (amorphous) carbon coexists with crystalline phases. The conspicuous peaks at 2θ ≈ 21.8°, 26.6°, 36.5°, 39.5°, and 54.8° match the lattice planes of α-quartz (JCPDS NO.99-0088 SiO_2_), confirming that the SiO_2_ inherited from the clay-based papermaking additives is thermally stable across the entire temperature window. The progressive sharpening of the graphitic diffuse peak at 26° with rising pyrolysis temperature evidences localized graphitization of the carbon matrix. Concomitantly, the marked attenuation of weak reflections at 30–35° and the CaCO_3_ characteristic peak at 900 °C reflects the thermal collapse of metastable mineral lattices and accompanying CO_2_ release. The resulting “quartz-embedded graphitized carbon” hybrid retains the high hardness and thermal stability of SiO_2_ while acquiring the electrical conductivity and π-electron delocalization capacity of graphitic crystallites, synergistically enhancing the biochar’s mechanical strength, chemical robustness, and performance in tetracycline adsorption and electron-transfer reactions.

#### 3.2.4. Specific Surface and Pore Size Distribution Results Analysis

**Figure 4:**
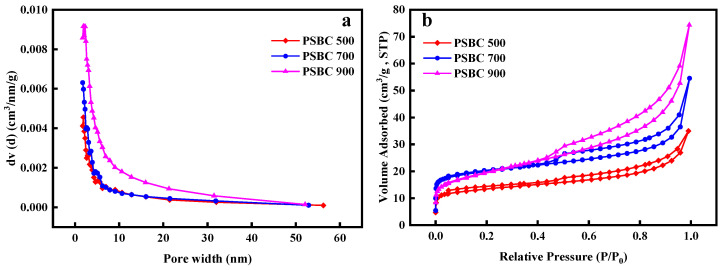
Pore size distribution of papermaking sludge biochars (PSBC 500, PSBC 700, and PSBC 900) (**a**) and N_2_ adsorption/desorption isotherms (**b**).

The paper sludge biochar’s (PSBC 500, PSBC 700, and PSBC 900) pore size distribution, N_2_ adsorption/desorption isotherms, specific surface area, and pore structure parameters are displayed in Figure 4 and Table 2. The isotherms of the paper sludge biochars (PSBC 500, PSBC 700, and PSBC 900) all fall into type IV according to the (IUPAC) classification criteria, as can be seen [15]. This suggests that the sludge developed a rich pore structure during high-temperature pyrolysis, which is primarily expressed as a mesoporous structure, confirming the findings of the SEM analysis.

PSBC 500, PSBC 700, and PSBC 900 had specific surface areas of 43.52, 64.55, and 79.53 m^2^/g, respectively, as indicated in Table 2. They also had pore diameters of 8.74, 8.95, and 9.86 nm and pore volumes of 0.041, 0.063, and 0.105 cm^3^/g. The micropores and mesopores of PSBC 900 were the most prevalent, and it is evident that the specific surface area, pore size, and pore volume of the paper sludge biochar increased as the pyrolysis temperature rose. This shows that the high temperature clearly contributed to the formation of porous biochar. This is because the high temperature caused bound water in paper sludge to fully precipitate, many organic components to continuously break down into small organic molecules, and the majority of organic matter, including cellulose, to volatilize through pyrolysis into volatile compounds, which led to the gradual formation of a porous biochar structure [16]. The optimal adsorption performance is attained when the adsorbent’s pore size is between 1.7 and three times that of the tetracycline pollutant, according to adsorption theory [14]. With dimensions of 1.41 nm for length, 0.46 nm for width, and 0.82 nm for height, the tetracycline molecule is a macromolecular adsorbate that can be efficiently absorbed by pores with radii between 1.2 and 4.23 nm [17]. According to the table, the pore sizes of the papermaking biochars (PSBC 500, PSBC 700, and PSBC 900) at various temperatures were 8.74, 8.95, and 9.86 nm, respectively. These pore sizes were located just inside the main channel, indicating that the paper sludge biochar had good tetracycline adsorption performance [9].

#### 3.2.5. Analysis of Fourier Transform Infrared Spectroscopy (FTIR) Results

**Figure 5:**
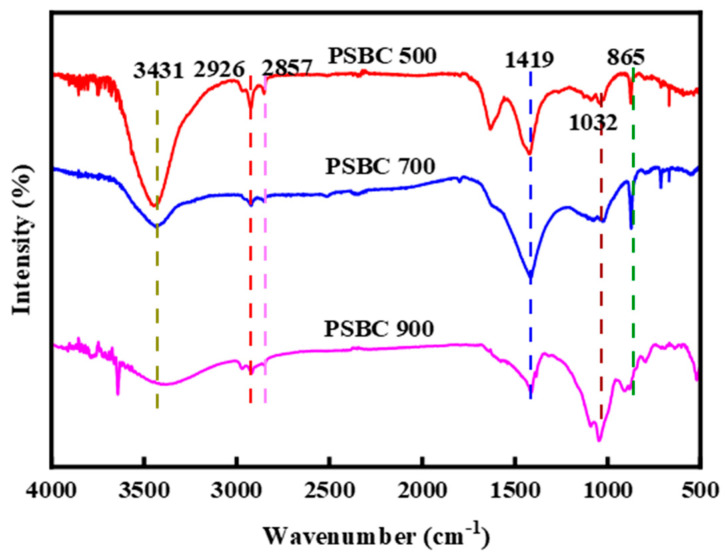
FTIR diagram for papermaking sludge biochars (PSBC 500, PSBC 700, and PSBC 900).

The FTIR profiles of biochars PSBC 500, PSBC 700, and PSBC 900 are shown in Figure 5.

The biochar made from papermaking sludge has nearly identical characteristic absorption peaks at three different temperatures, as shown in Figure 5. There is a stretching vibration peak for the hydroxyl group (OH) at 3431 cm^−1^ and C–H stretching vibrations are represented by 2857 cm^−1^, while C=C stretching vibrations are shown by 1419 cm^−1^. The presence of an olefinic C–H out-of-plane bending vibration at 865 cm^−1^ suggests a high functional group content that is generally constant throughout a range of temperatures, whereas the peak at 1032 cm^−1^ corresponds to C–O stretching vibrations [18].

The persistent C=C stretching vibration at 1419 cm^−1^ evidences the structural integrity of the aromatic backbone, confirming that π–π stacking constitutes the principal adsorption mechanism. The concurrent signals at 1032 cm^−1^ (C–O of phenolic –OH and ether bridges) and 865 cm^−1^ (olefinic C–H) demonstrate the coexistence of electrostatic attraction and surface complexation in the tetracycline-binding process. The broad band centered at 3431 cm^−1^, assigned to hydroxyl (–OH) functionalities, identifies these groups as the dominant proton donors capable of forming hydrogen bonds with the amide and phenolic moieties of tetracycline. As the pyrolytic temperature is raised from 500 °C to 900 °C, progressive dehydration and condensation reactions diminish the –OH population, enabling π–π interactions to prevail. This evolution in surface chemistry rationalizes the superior tetracycline uptake capacity exhibited by PSBC-900.

#### 3.2.6. Analysis of Raman Spectroscopy Results

**Figure 6:**
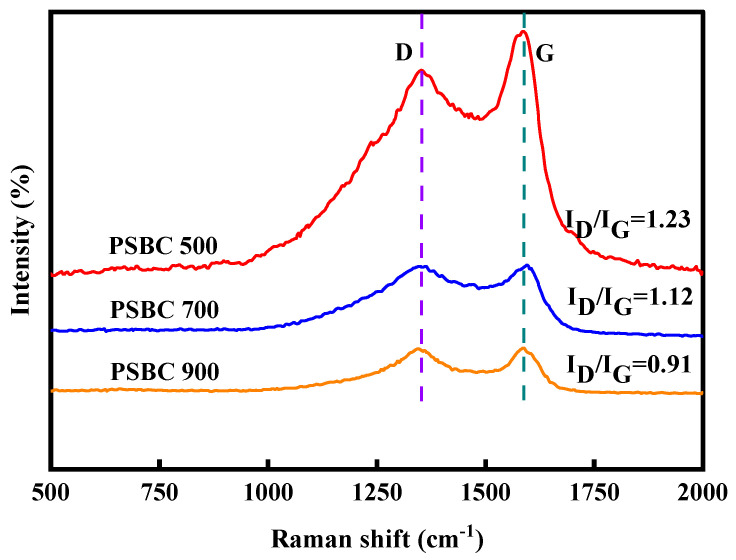
Raman diagram for papermaking sludge biochars PSBC 500, PSBC 700, and PSBC 900.

Raman spectroscopy was used to further evaluate the graphitization degree of biochar. The results are displayed in Figure 6, where the characteristic peaks D and G of PSBC 500, PSBC 700, and PSBC 900 were found at 1337 and 1599 cm^−1^, 1351 and 1596 cm^−1^, and 1345 and 1598 cm^−1^, respectively. The G peak indicates the degree of carbonization of biochar, while the D peak indicates the degree of defects of biochar [19]. The degree of graphitization of biochar is indicated by the intensity ratio (I_D_/I_G_) of the D peak to the G peak, which is inversely proportional to its ratio [20]. The PSBC 500, PSBC 700, and PSBC 900 biochars had calculated I_D_/I_G_ values of 1.23, 1.12, and 0.79, respectively. This suggests that the paper sludge biochars have higher degrees of graphitization, which is consistent with the XRD results, which show that PSBC 900 has a higher degree of graphitization. This suggests that high temperatures are more conducive to the formation of the porous structure, which results in a higher degree of graphitization.

### 3.3. Analysis of Adsorption Results

#### 3.3.1. Effect of Biochar Dosage on Adsorption

**Figure 7:**
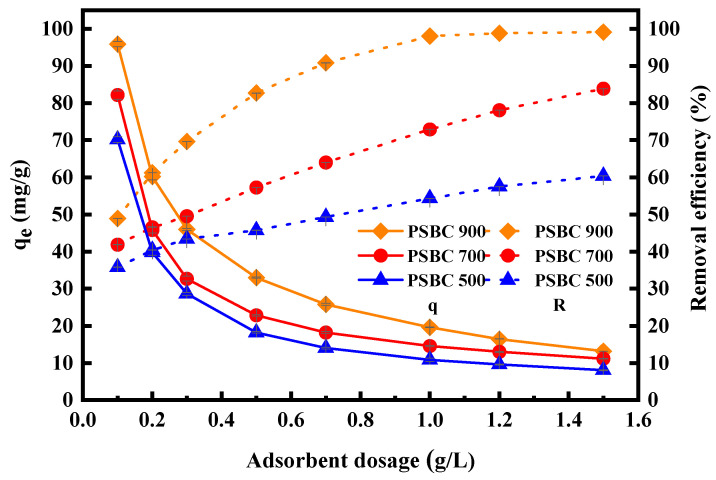
Effect of dosage on adsorption of tetracycline by papermaking sludge biochars PSBC 500, PSBC 700, and PSBC 900.

In order to investigate the impact of varying dosages of paper sludge biochars (PSBC 500, PSBC 700, and PSBC 900) on the adsorption of tetracycline, the following dosages were set up in this study: 0.10, 0.20, 0.30, 0.50, 0.70, 1.00, 1.20, and 1.50 g/L. Figure 7 displays the outcomes of the study.

The removal rate of tetracycline by PSBC 500 increased from 35.78% to 60.34% as the dosage of paper sludge biochar was increased from 0.10 g/L to 1.50 g/L. At the same time, the adsorption amount of tetracycline by PSBC 500 decreased from 70.17 mg/g to 8.02 mg/g. The removal rate of tetracycline by PSBC 700 increased from 41.90% to 83.91%, while the amount of tetracycline adsorbed by PSBC 700 decreased from 82.16 mg/g to 11.15 mg/g. The removal rate of tetracycline by PSBC 900 increased from 48.90% to 99.15%, while the amount of tetracycline adsorbed by PSBC 900 decreased from 95.88 mg/g to 13.18 mg/g.

In conclusion, the adsorption and removal of tetracycline from wastewater solutions can be influenced by the dose of three distinct temperature-prepared biochars (PSBC 500, PSBC 700, and PSBC 900), with generally comparable but marginally varied trends. Tetracycline removal rates for PSBC 500, PSBC 700, and PSBC 900 were 54.39%, 72.19%, and 98.06%, respectively, at a dosage of 1.00 g/L. Adsorption capacities reached 10.85 mg/g, 14.57 mg/g, and 19.86 mg/g, respectively. When the dosage was increased, PSBC 900’s removal rates and adsorption capacities displayed a flat trend. Following that, when the dosage was raised, PSBC 900’s tetracycline removal rate and adsorption capacity displayed a flat trend, whereas PSBC 500 and 700’s tetracycline removal rates continued to show an increasing trend, albeit with a smaller magnitude, and their adsorption capacities tended to be stable. During the experiment, the controlling variable was the initial mass concentration of tetracycline-simulated wastewater. The wastewater solution’s higher tetracycline content at a lower biochar dosage encouraged tetracycline molecules to rapidly occupy the biochar’s adsorption active sites through adsorption and diffusion, reaching equilibrium over time. The adsorption sites on the surface of biochar increased in proportion to the dosage of biochar, which raised the rate at which tetracycline was removed. Nevertheless, even with constant increases in the dosage of biochar, extra active sites would be produced after adsorption saturation, and the adsorption impact would not be appreciably enhanced. The ideal dosage of biochar for paper sludge was determined to be 1.00 g/L by combining the removal effect with economics.

It is evident that the adsorption performance of paper sludge biochar on tetracycline is directly proportional to the temperature of pyrolysis preparation when comparing the adsorption removal rate and adsorption capacity of the biochar under the same conditions in the order of PSBC 900 > PSBC 700 > PSBC 500.

#### 3.3.2. Effect of Reaction Time on Adsorption

**Figure 8:**
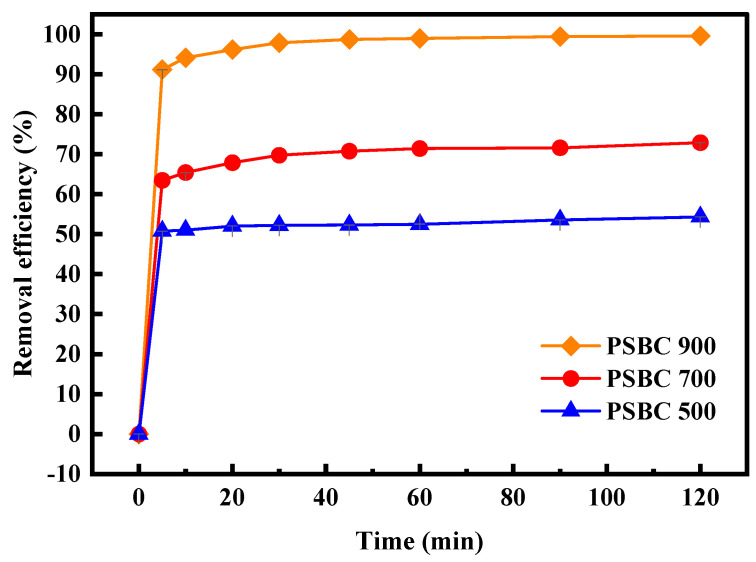
Effect of adsorption time on adsorption of tetracycline by papermaking sludge biochars PSBC 500, PSBC 700, and PSBC 900.

In order to examine the impact of varying adsorption times on the adsorption performance of tetracycline on paper sludge biochars (PSBC 500, PSBC 700, and PSBC 900), the adsorption system’s reaction time was set at 5, 10, 20, 30, 45, 60, 90, and 120 min. The experimental results are displayed in Figure 8.

As demonstrated in Figure 8, the paper sludge biochars (PSBC 500, PSBC 700, and PSBC 900) displayed the same trend over time. During the first 10 min of the reaction, the removal rates of PSBC 500, PSBC 700, and PSBC 900 were 51.02%, 65.42%, and 94.12%, respectively. All of them indicated a rapidly increasing trend in the tetracycline removal rate. The primary cause of this is that, initially, tetracycline pollutants in the wastewater solution quickly occupied the adsorption sites on the surface of the paper sludge biochar through physical adsorption. At the same time, the system’s higher initial mass concentration of tetracycline at the beginning stage resulted in an enhanced driving force to accelerate the adsorption rate [21]. Tetracycline pollutants occupied a large number of active sites on the surface of the biochar of the paper sludge, and at the same time, a large amount of tetracycline in the system was adsorbed, resulting in the small concentration of tetracycline, which in turn reduced the adsorption driving force, leading to the equilibrium state. This is why the removal rate of tetracycline by PSBC 500, PSBC 700, and PSBC 900 gradually leveled off after 90 min of reaction and then continued to adsorb until 120 min, when it remained essentially unchanged. The equilibrium state was reached when the adsorption driving force was decreased [22]. In order to make the reaction more suitable, 120 min was ultimately chosen as the adsorption equilibrium duration. The removal efficiencies at equilibrium were 54.32%, 72.91%, and 99.54%, respectively.

#### 3.3.3. Effect of Initial Mass Concentration of Tetracycline on Adsorption

**Figure 9:**
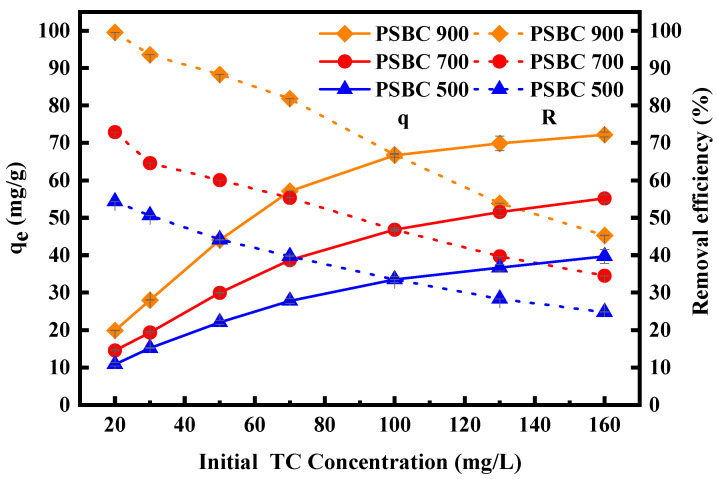
Effect of initial mass concentration of tetracycline on adsorption of tetracycline by papermaking sludge biochars PSBC 500, PSBC 700, and PSBC 900.

In order to examine the impact of the initial mass concentration of the tetracycline solution on the removal of tetracycline by adsorption on the biochars of paper sludge (PSBC 500, PSBC 700, and PSBC 900), we decided to set up the wastewater solution with tetracycline mass concentrations of 20, 30, 50, 70, 100, 130, and 160 mg/L. The experiments’ outcomes are displayed in Figure 9.

As illustrated in Figure 9, as the mass concentration of tetracycline in the reaction system increased from 20 mg/L to 160 mg/L, the removal rate of tetracycline by PSBC 500 gradually decreased from 54.32% to 24.79% and the amount of tetracycline by PSBC 500 gradually increased from 10.85 mg/g to 39.67 mg/g; the removal rate of tetracycline by PSBC 700 gradually decreased from 72.91% to 34.47%, while the amount of tetracycline adsorbed by PSBC 700 gradually increased from 14.56 mg/g to 55.15 mg/g; the tetracycline removal rate by PSBC 900 gradually decreased from 99.54% to 45.26%, while the amount of tetracycline adsorbed by PSBC 900 gradually increased from 19.87 mg/g to 72.22 mg/g.

In conclusion, the biochars produced at three different temperatures (PSBC 500, PSBC 700, and PSBC 900) demonstrated a decreasing trend in tetracycline adsorption and removal rates in the wastewater solution as the initial mass concentration of tetracycline increased. At the same time, the adsorption capacity gradually increased and then leveled off. This is because the pressure between the biochar and the increase in tetracycline mass concentration is higher, which causes a considerable increase in the driving force between the two. As a result, the higher tetracycline molecules continue to occupy the excess active adsorption sites in the paper sludge biochar, increasing the adsorption capacity [23]. Because the dosage of biochar is controlled, the rate at which the total adsorption capacity increases slows down until it stops rising because the excess active sites in the paper sludge biochar are occupied and saturated by a large number of tetracycline molecules. The best adsorption performance of PSBC 900 was confirmed when it was discovered that, for tetracycline at various initial mass concentrations, its adsorption removal rate and adsorption capacity were superior to those of PSBC 700, which were superior to those of PSBC 500.

#### 3.3.4. Effect of Solution pH on Adsorption

**Figure 10:**
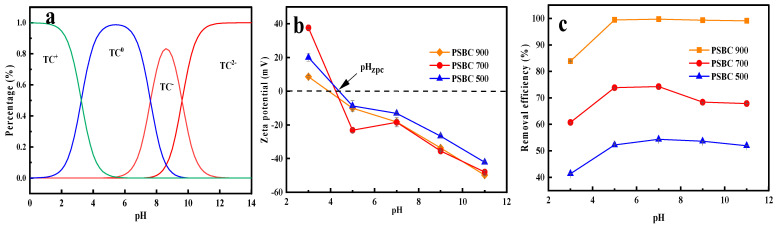
(**a**) Molecular morphological distribution of different pH tetracyclines; (**b**) curves of zeta potential of papermaking sludge biochars PSBC 500, PSBC 700, and PSBC 900 with pH; (**c**) effect of initial pH of solution on adsorption of tetracycline in papermaking sludge biochars PSBC 500, PSBC 700, and PSBC 900.

The effect of paper sludge biochars (PSBC 500, PSBC 700, and PSBC 900) on the adsorption of tetracycline under the conditions of tetracycline solution in wastewater with varying pH values was investigated in this study using pH values of 3.0, 5.0, 7.0, 9.0, and 11.0. The experiments’ outcomes are displayed in Figure 10.

In addition to somewhat influencing the solubility of paper sludge biochar, the pH of the reaction system solution also modifies the adsorbent’s surface charge properties and its morphology, which alters the electrostatic interactions (attraction or repulsion) between the adsorbate and the adsorbent mass and influences the adsorption effect. Tetracycline removal by PSBC 500 increased gradually from 41.36% at a pH equal to 3.0, to 54.32% at a pH equal to 7.0, then decreased slightly to 51.91% at a pH equal to 11.0. As the pH was raised from 3.0 to 11.0, as shown in Figure 10c, PSBC 700’s tetracycline elimination rate gradually increased from 60.73% at a pH equal to 3.0, to 74.25% at pH equal to 7.0, then decreased to 67.88% at pH equal to 11.0. Similarly, PSBC 900’s tetracycline removal rate increased gradually from 83.86% at pH 3.0 to 99.69% at pH 7.0 before exhibiting a flat trend as the pH increased. Simultaneously, the clearance rate at pH 7.0 was nearly identical to that at pH 7.0 during the blank control experiment. Its highest removal rates during this time were 54.32%, 74.25%, and 99.78%, in that order. Therefore, it was determined that when the solution’s original pH was 7.0 or left unaltered, PSBC 500, PSBC 700, and PSBC 900 had the best adsorption effect on tetracycline.

Tetracycline is an amphoteric chemical with three dissociation constants that can exhibit several states in aqueous settings with varying pH values: molecule (TC^0^), cation (TC^+^), and anion (TC^−^ and TC^2−^) [24]. Figure 10a displays the distribution of tetracycline’s molecular morphology at various pH levels, while Figure 10b displays the zero potential points (pHpzc) of PSBC 500, PSBC 700, and PSBC 900, which were found to be 4.41, 4.22, and 3.93, respectively. The point of zero charge (pHpzc) is defined as the pH at which the net surface charge of the biochar adsorbent is zero, and it is widely employed to evaluate the sign and magnitude of surface charges under varying solution chemistries. Electrostatic interactions between the biochar surface and pollutant species are strongly modulated by this parameter. At pH > pHpzc, the biochar surface is deprotonated and carries a net negative charge, favoring the adsorption of cationic contaminants via electrostatic attraction; conversely, at pH < pHpzc, surface protonation yields a net positive charge, thereby promoting the uptake of anionic species. Given that the primary species of tetracycline in the reaction system at pH 3.3 is protonated TC (TC^+^), which is positively charged, and that the biochar is also positively charged at this time, it is evident from Figure 10c that the adsorption of tetracycline by two types of biochar in the solution with a pH between 3.0 and 7.0 is positively proportional to the pH’s positive charge; therefore, its primary force is electrostatic repulsion, which lessens the paper sludge biochar’s adsorption impact [8]. Additionally, the primary tetracycline type, TC^0^, exhibits neutral overall performance when the pH value of the system falls between 3.3 and 7.7. During this period, the biochar’s positive charge is diminished, and the negative charge is progressively raised to display a negative charge. As a result, the two have less electrostatic repulsion and the adsorption effectiveness rises. The adsorption and removal impact of tetracycline by PSBC 500 and PSBC 700 gradually weakened when the pH value of the reaction system was between 7.7 and 9.0, as TC^0^ changed to TC^−^, displaying negative charge, and there was electrostatic repulsion between the two; however, the adsorption capacity of PSBC 900 remained robust and did not exhibit a declining trend, indicating that PSBC 900 could be employed in the reaction system at a pH of 7.7–9.0. and with tetracycline molecules at a pH of 7.7–9.0. Other interaction forces exist in addition to electrostatic interactions. Although the adsorption efficiencies of PSBC 500, PSBC 700, and PSBC 900 were not considerably reduced, the negative charge on the surface of the tetracycline molecule changed from TC^−^ to TC^2−^ biochar when the pH of the system was more than 9.7. This resulted in increased electrostatic repulsion. It also implies that there are additional interacting forces at work and that electrostatic interactions are not their primary adsorption mechanism. Similar findings were confirmed in the other literature [8].

#### 3.3.5. Effect of Different Ion Concentrations on Adsorption

**Figure 11:**
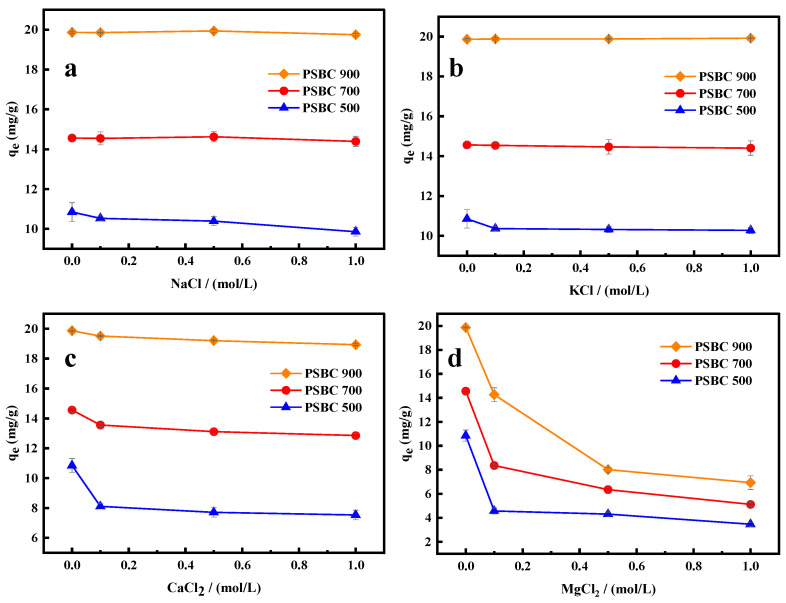
Effects of different cation concentrations on the adsorption of tetracycline by papermaking sludge biochars PSBC 500, PSBC 700, and PSBC 900. (**a**) NaCl concentrations on the adsorption of tetracycline, (**b**) KCl concentrations on the adsorption of tetracycline, (**c**) Ca_2_Cl concentrations on the adsorption of tetracycline, (**d**) MgCl_2_ concentrations on the adsorption of tetracycline.

In practice, a high salt content in the tetracycline effluent may compete with the tetracycline molecules for the active sites on the sludge biochar’s surface during the adsorption process, weakening the sludge biochar’s ability to remove tetracycline from the reaction system [6]. Since salt ions like sodium, potassium, calcium, and magnesium are frequently present in actual wastewater, the current study simulated the configuration of various salt ion concentrations, 0, 0.10, 0.50, and 1.00 mol/L of NaCl, KCl, CaCl_2_, and MgCl_2_ solutions added to tetracycline solution at a concentration of 20 mg/L. This allowed the researchers to examine the impact of varying salt ion concentrations on the adsorption of tetracycline in paper sludge biochar adsorption solutions. Figure 11 displays the exact experimental results. In the range of Na^+^ (0–1.0 mol/L), the adsorption capacities of PSBC 500, PSBC 700, and PSBC 900 varied between 9.86 and 10.58 mg/g, 14.40–14.62 mg/g, and 19.75–19.94 mg/g, respectively, according to the comparison in Figure 11. Meanwhile, in the range of K^+^ (0–1.0 mol/L), the adsorption capabilities of PSBC 500, PSBC 700, and PSBC 900 varied between 10.27 and 10.85 mg/g, 14.41–14.56 mg/g, and 19.87–19.92 mg/g, with minimal overall changes. The figure also shows that the effects of Na^+^ and K^+^ on tetracycline adsorption were almost at the same level, suggesting that these two elements had virtually little effect on the adsorption capacity of tetracycline adsorbed on paper sludge biochar. In the range of Ca^2+^ (0~1.0 mol/L), the adsorption capacities of PSBC 500, PSBC 700, and PSBC 900 varied between 10.85~8.11~7.71~7.54 mg/g and 4.56~13.56~13.11~12.58 mg/g, respectively. The image reduction was minimal, suggesting a comparatively weak inhibition of adsorption by calcium ions. In the range of Mg^2+^ (0–1.0 mol/L), however, the adsorption capacities of PSBC 500, PSBC 700, and PSBC 900 were 10.85~4.56~4.30~3.46 mg/g, 14.56~8.35~6.36~5.11 mg/g, and 19.87~14.26~8.01~6.93 mg/g, respectively. The graph also shows a downward trend, suggesting that Mg^2+^ has a comparatively potent inhibitory impact.

The experiments revealed that the high concentration of 1 mol/L Na^+^ and K^+^ had no effect on the adsorption of tetracycline by biochar, indicating that other interaction forces (such as hydrogen bonding and π–π electron–donor interactions) existed in the system of biochar [6]. Under normal circumstances, monovalent Na^+^ and K^+^ bind to the active site of tetracycline and play a certain inhibitory effect due to the electrostatic interaction. The two should exhibit the same inhibition results because of the stronger electrostatic interaction between divalent Ca^2+^ and Mg^2+^ and the paper sludge biochar. However, experiments revealed that the divalent Mg^2+^ had a greater inhibitory effect because the high valence resulted in more positive charges, which encouraged more active sites to bind with them and decreased the binding capacity of the active sites on the paper sludge biochar’s surface to tetracycline, resulting in a decrease in adsorption performance. Relevant research reports indicate that divalent Mg^2+^ can complex on the surface of biochar, creating a water layer that is adhered to the surface. This may be the cause of the decrease in tetracycline adsorption by divalent Mg^2+^, as it prevents tetracycline from binding to the active sites on the surface of biochar [25]. The experimentally generated paper sludge biochar has a considerable selective adsorption capacity for tetracycline, as demonstrated by the fact that, even at high concentrations, the other three ions show no discernible inhibition, with the exception of the strong inhibition of Mg^2+^.

### 3.4. Adsorption Modeling Analysis

#### 3.4.1. Adsorption Kinetics

**Figure 12:**
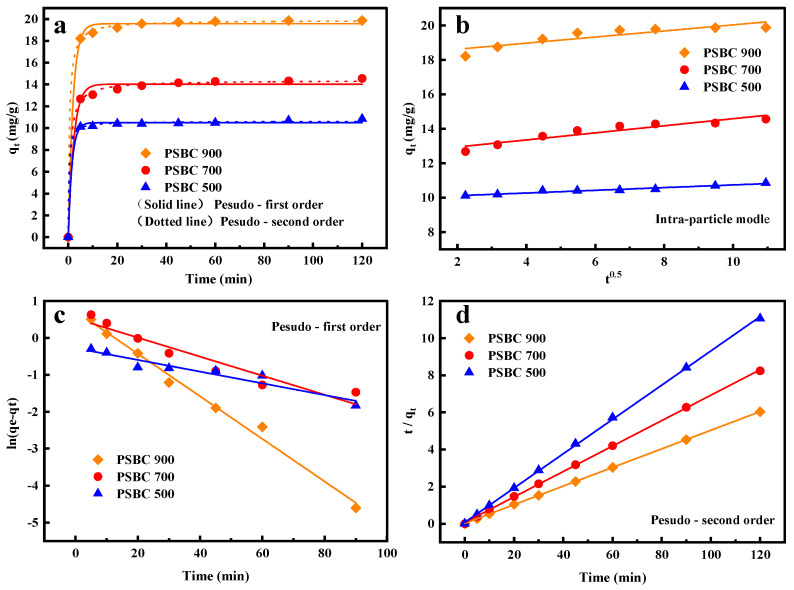
(**a**) Papermaking sludge biochars, PSBC 500, PSBC 700, and PSBC 900, kinetic model plots, (**b**) intra-particle diffusion model plots, (**c**) quasi-primary kinetic model linear fit plots, and (**d**) quasi-secondary kinetic model linear fit plots.

In order to analyze the kinetic adsorption process of paper sludge, the kinetic experimental data of tetracycline adsorption by paper sludge biochars (PSBC 500, PSBC 700, and PSBC 900) were fitted using quasi-primary kinetic models, quasi-secondary kinetic models, and intra-particle diffusion models. Figure 12 displays the fitting results, while Table 3 displays the fitting parameters.

The fitted experimental data in Table 3 were derived from the curve and two linear fits in Figure 12. Table 3 shows that the quasi-secondary kinetic correlation coefficients (R^2^) of PSBC 500, PSBC 700, and PSBC 900 are 0.9997, 0.9995, and 0.9999, respectively. These correlation coefficients are superior to those of the quasi-primary kinetic model (R^2^ = 0.9202, 0.9154, and 0.9992). The fitting equations yielded theoretical adsorption amounts of 10.82, 14.58, and 19.94 mg/g, while the actual adsorption amounts were PSBC 500, PSBC 700, and PSBC 900, respectively. According to the fitted equations, the predicted adsorption amounts of PSBC 500, PSBC 700, and PSBC 900 were 10.82, 14.58, and 19.94 mg/g, respectively. These values were more in line with the actual adsorption amounts of 10.85, 14.56, and 19.87 mg/g. It suggests that chemisorption is the primary process regulating the rate and that the adsorption of tetracycline from wastewater by paper biochar in the reaction system is more pertinent to the quasi-secondary kinetic model [24].

While the quasi-primary equation describes the first stage of the adsorption process in the solid–liquid phase [21], which is frequently characterized by physical adsorption [26], the quasi-secondary equation includes a variety of adsorption mechanisms, including intra-particle diffusion and electron sharing or transfer, liquid film diffusion, surface adsorption, and other adsorption processes [9]. The straight line fitted to the paper sludge biochar on wastewater tetracycline in the reaction system does not pass through the origin, as can be seen based on the theory of intra-particle diffusion model, as illustrated in Figure 12b. This suggests that the tetracycline adsorption rate of paper sludge biochar adsorption involves a number of processes, of which particle diffusion is only one. Other factors may include membrane diffusion and chemical reactions, etc. [6].

#### 3.4.2. Isotherm Analysis

**Figure 13:**
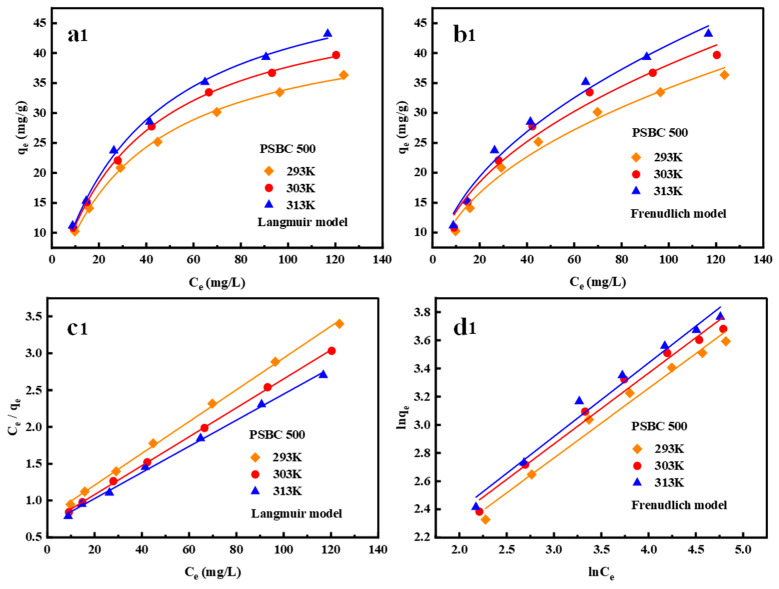

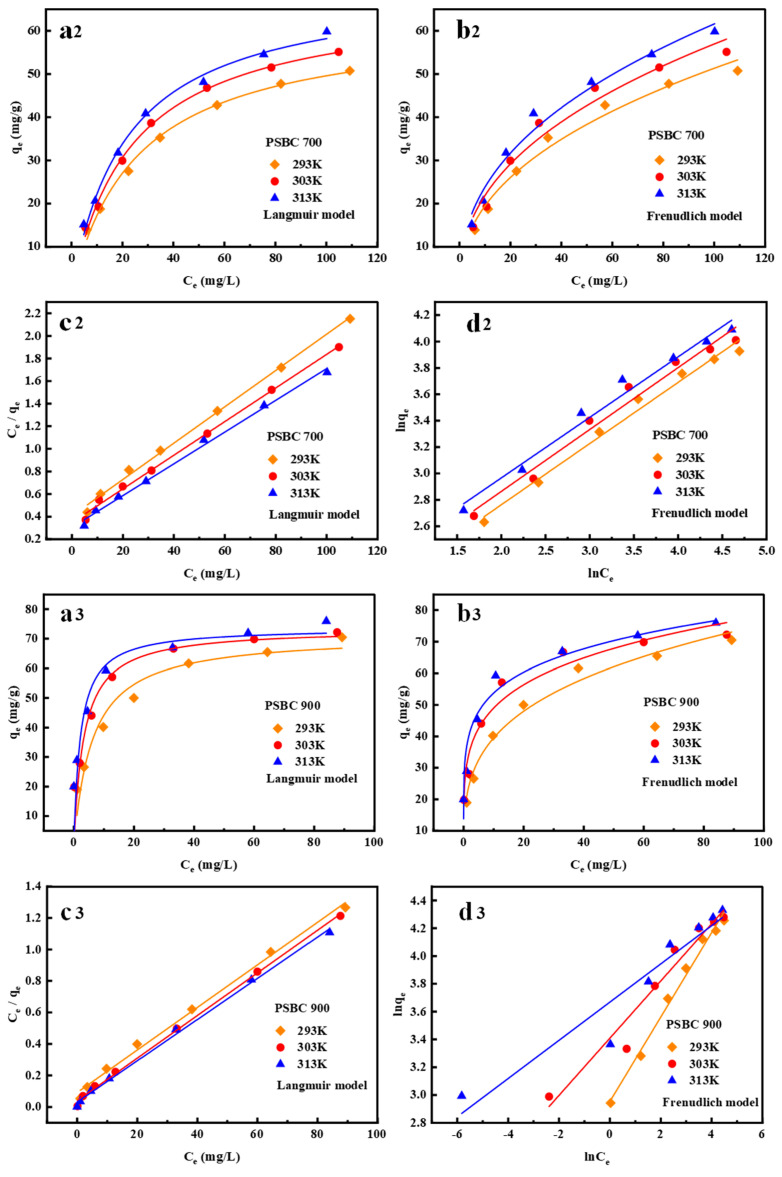
Papermaking sludge biochars PSBC 500, PSBC 700, and PSBC 900 (**a1**–**a3**) Langmuir model plot, (**b1**–**b3**) Freundlich model plot, (**c1**–**c3**) Langmuir model linear fit plot, and (**d1**–**d3**) Freundlich model linear fit plot.

In order to analyze the isothermal adsorption mechanism of paper sludge, the Langmuir and Freundlich isotherm models were used to match the isothermal experimental data of tetracycline adsorption on paper sludge biochars (PSBC 500, PSBC 700, and PSBC 900). Table 4 displays the fitting parameters and Figure 13 displays the fitting results. The Langmuir model R^2^ values of biochars PSBC 500, PSBC 700, and PSBC 900 at temperatures of 20, 30, and 40 °C were 0.9974–0.9994, 0.9954–0.9969, and 0.9951–0.9985 higher than the Freundlich model R^2^ values of 0.9756–0.9782, 0.9169–0.9893, and 0.9196–0.9886 under the same conditions, according to the curves and linear fitting results in Figure 13. The adsorption process of tetracycline by paper sludge biochar was more relevant to the Langmuir model, and the homogeneous adsorption of the mono-molecular layer in the adsorption process was predominant, according to the Freundlich model R^2^ values of 0.9756–0.9782, 0.9169–0.9893, and 0.9196–0.9886 [9]. While both the qmax and KL parameters demonstrated a positive relationship with the reaction temperature, suggesting that raising the temperature is favorable for adsorption, the KL values ranged between 0 and 1, suggesting that the adsorption process of tetracycline by biochar is a favorable process [21].

The Freundlich’s R^2^ of biochars PSBC 500, PSBC 700, and PSBC 900 were all greater than 0.90, which indicated that the adsorption of tetracycline by paper biochar was also partially inhomogeneous at the active site [27]. The value of 1/n was less than 0.5, which indicated that the paper biochar could adsorb tetracycline in wastewater systems better [14]. The relationship between the paper biochar constants K_F_ was PSBC 900 > PSBC 700 > PSBC 500, which indicated that the paper sludge biochar (PSBC 900) prepared under a high-temperature pyrolysis environment had better adsorption performance. This is consistent with the conclusion of the previous adsorption analysis.

#### 3.4.3. Thermodynamic Analyses

In order to analyze the thermodynamic behavior of paper sludge, thermodynamic modeling was used to fit the thermodynamic experimental data of tetracycline adsorption on paper sludge biochars (PSBC 500, PSBC 700, and PSBC 900). Table 5 displays the precise parameters that were fitted.

The adsorption was spontaneous at the experimental temperature, as indicated by the negative ∆G^θ^ values of biochars PSBC 500, PSBC 700, and PSBC 900. The absolute value of ∆G^θ^ was proportional to the temperature, confirming that raising the temperature was favorable for adsorption and that the tetracycline molecules could quickly travel to the adsorption sites on the biochar’s surface and bind to them [21]. The energy change brought about by the interaction between the adsorbed species and the adsorbent is represented by the enthalpy change (∆H^θ^) [26]. At every temperature, the three biochars’ ∆H^θ^ values were positive, suggesting that this is a heat-absorbing adsorption reaction [6]. The positive values of entropy change (∆S^θ^) for all three biochars demonstrate the biochars’ high affinity for tetracycline during the reaction process [17]. Entropy change is a measure of repulsive or binding forces in a system that is connected to the spatial arrangement of the adsorbent interfaces. In conclusion, the reaction system exhibits spontaneous heat absorption and entropy growth during the tetracycline adsorption process by paper sludge biochar.

### 3.5. Adsorption and Regeneration Analysis

**Figure 14:**
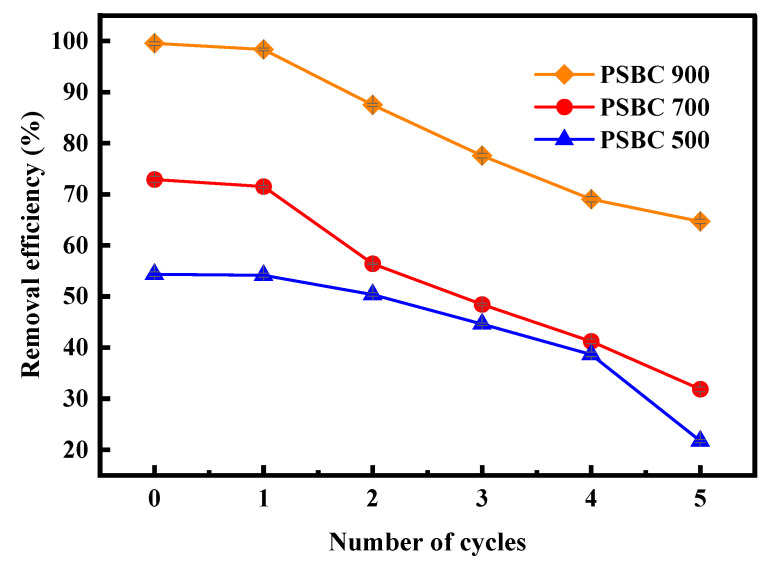
Recycling of papermaking sludge biochars PSBC 500, PSBC 700, and PSBC 900.

Five regeneration cycle tests were used to further examine the regeneration and reuse properties of the adsorbents in order to assess the potential of adsorbent materials in real-world scenarios. Figure 14 presents the experimental results, which show that the adsorption capacity of paper sludge biochars (PSBC 500, PSBC 700, and PSBC 900) on tetracycline gradually decreased as regeneration times increased. The amount of tetracycline that was adsorbed on PSBC 500 decreased from 10.85 mg/g to 4.33 mg/g, and the removal rate decreased from 54.32% to 21.68%; tetracycline’s adsorption by PSBC 700 dropped from 14.56 mg/g to 6.36 mg/g, resulting in a decrease in removal rate from 72.91% to 31.84%; similarly, tetracycline’s adsorption by biochar PSBC 900 dropped from 19.87 mg/g to 12.92 mg/g, resulting in a decrease in removal rate from 99.54% to 64.68%. These results all demonstrated a distinct downward trend that is not negligible. Tetracycline’s partial desorption during the recovery phase may be the primary cause of this, since it restricts the drug’s ability to bind to the biochar’s active sites [20].

Paper sludge, a solid waste that comes from a variety of sources, is the raw material needed to make biochar in this study. Because the preparation procedure is low-cost, it falls into the waste resource-utilization category. Therefore, to improve the adsorption performance when contemplating reuse, a specific amount of fresh paper sludge biochar can be added. The potential applications of sludge biochar as a cost-effective and reusable adsorbent for the treatment of antimicrobial wastewater are numerous.

## 4. Conclusions

Papermaking sludge was successfully converted into silicon-rich biochar (PSBC) with a high specific surface area via oxygen-limited pyrolysis at temperatures ranging from 500 °C to 900 °C. The results demonstrated that increasing pyrolytic temperatures concurrently enhanced pore development, graphitization degree, and surface hydrophilicity, leading to a linear increase in tetracycline (TC) adsorption capacity, with PSBC 900 exhibiting optimal performance. The removal efficiency of the as-prepared adsorbent increased progressively from 83.36% at pH 3.0 to 99.69% at pH 7.0. Notably, coexisting cations such as Na^+^, K^+^, and Ca^2+^ exerted negligible influence on TC adsorption, whereas Mg^2+^ exhibited a discernible inhibitory effect.

Adsorption kinetics, isotherms, and thermodynamic modeling revealed that TC uptake by PSBC across various temperatures is a spontaneous, endothermic, and entropy-increasing process, governed by monolayer chemisorption. This was evidenced by a negative Gibbs free energy change ($∆G^{\theta }$ < 0), positive enthalpy change ($∆H^{\theta }$ > 0), and positive entropy change ($∆S^{\theta }$ > 0), with the adsorption behavior well-fitted by the pseudo-second-order kinetic model and the Langmuir isotherm model (R^2^ > 0.99).

Furthermore, PSBC 900 maintained a removal efficiency exceeding 60% after five consecutive regeneration cycles, underscoring its favorable reusability and economic viability. These findings highlight the promising potential of PSBC 900 as a sustainable and efficient adsorbent for the remediation of antibiotic-contaminated wastewater.

## Figures and Tables

**Table 1:** Elemental analysis of papermaking sludge biochars (PSBC 500, PSBC 700, and PSBC 900): elemental composition (C, H, O, N, and mass fractions) and atomic ratios.

Sample	Elemental Composition (%)				Atomic Ratio		
	C	H	N	O	H/C	O/C	(N+O)/C
PSBC 500	23.45	1.21	0.48	21.86	0.05	0.93	0.95
PSBC 700	15.72	0.59	0.31	16.89	0.03	1.07	1.09
PSBC 900	13.51	0.37	0.18	15.96	0.02	1.18	1.19

**Table 2:** Specific surface area and pore structure parameters of papermaking sludge biochars PSBC 500, PSBC 700, and PSBC 900.

Sample	S_BET_ (m^2^/g)	V_total_/(cm^3^/g)	D_aver_/nm
PSBC 500	43.52	0.041	8.74
PSBC 700	64.55	0.063	8.95
PSBC 900	79.53	0.105	9.86

**Table 3:** Parameters of adsorption kinetic model.

Biochar	q_e_, exp (mg/g)	Pseudo-First-Order			Pseudo-Second-Order			Intra-Particle-Model		
		q_e_, cal (mg/g)	K_1_ (min^−1^)	R^2^	q_e_, cal (mg/g)	K_2_ (g/mg/min)	R^2^	C	K_id_	R^2^
PSBC 500	10.85	10.49	0.0158	0.9202	10.82	0.1141	0.9997	9.95	0.078	0.9544
PSBC 700	14.56	14.02	0.0258	0.9154	14.58	0.0665	0.9995	12.52	0.205	0.8899
PSBC 900	19.87	19.56	0.0577	0.9902	19.89	0.1059	0.9999	18.25	0.177	0.7843

**Table 4:** Papermaking sludge biochars PSBC 500, PSBC 700, and PSBC 900 Langmuir model and Freundlich model-related parameters.

Biochar	T(K)	Langmuir Model			Freundlich Model		
		K_L_ (L/mg)	q_m_ (mg/g)	R^2^	K_F_ (mg/g)	1/n	R^2^
PSBC 500	293	0.027	46.34	0.9988	3.60	0.494	0.9774
	303	0.028	50.94	0.9994	3.88	0.487	0.9756
	313	0.026	56.40	0.9974	3.86	0.473	0.9782
PSBC 700	293	0.039	62.19	0.9968	6.26	0.464	0.9893
	303	0.042	67.16	0.9969	6.85	0.469	0.9536
	313	0.046	71.17	0.9954	7.75	0.459	0.9169
PSBC 900	293	0.148	73.91	0.9951	19.18	0.303	0.9886
	303	0.343	74.13	0.9985	30.24	0.205	0.9536
	313	0.419	76.39	0.9973	39.19	0.137	0.9196

**Table 5:** Papermaking sludge biochars, PSBC 500, PSBC 700, and PSBC 900, thermodynamic data sheet.

Biochar	T(K)	$∆G^{\theta }$ (KJ/mol)	$∆H^{\theta }$ (KJ/mol)	$∆S^{\theta }$ (J/(mol·K))
PSBC 500	293	−1.24	5.39	14.37
	303	−0.92		
	313	−0.96		
PSBC 700	293	−0.69	8.13	30.038
	303	−0.94		
	313	−1.29		
PSBC 900	293	−3.23	33.50	125.47
	303	−4.56		
	313	−5.74		

## Data Availability

Original data were generated for this article but are not in a publicly available repository. Data are contained within the article or Supplementary Materials.

## Supplementary Materials

The following supporting information can be downloaded at: https://www.mdpi.com/article/10.3390/toxics13121050/s1, Figure S1: TG-DTG diagram of paper sludge.
