# Supplementary material for: Preparation of Biochar from Papermaking Sludge and Its Adsorption Characteristics for Tetracycline
Source: Toxics. 2025 Dec 4;13(12):1050. doi: 10.3390/toxics13121050 (PMC12737266; doi:10.3390/toxics13121050)
Supplement: Supplementary file 1 [file toxics-13-01050-s001.zip › toxics-3984508-supplementary.pdf]

**Supplementary Information for:**

# Preparation of Biochar from Papermaking Sludge and Its Adsorption Characteristics for Tetracycline

Jiayu Niu [1,2,3], Siyuan Fan [1] and Zhenjun Wu [1,2,3,*]

1 School of Environmental Engineering, Henan University of Technology, Zhengzhou 450001, China

2 Zhengzhou International Cooperation Base for Science and Technology on Carbon Neutrality of Organic Solid Waste Conversion, Zhengzhou 450001, China

3 Henan International Joint Laboratory of Environmental Pollution Remediation and Grain Quality Security, Zhengzhou 450001, China

* Correspondence: wuzhenjun@haut.edu.cn

The first stage ranges from 30°C to 150°C, during which the weight loss of the paper sludge is approximately 1%, primarily due to the volatilization of free water in the sludge, along with intermolecular bound water. Volatile organic matter in the sludge precipitates during this stage. The second stage occurs around 150~650°C, characterized by a sharp increase in the rate of weight loss. Peaks in weight loss are observed at approximately 350°C for papermaking sludge, with weight loss proportions of approximately 43%. During this period, macromolecular organic matter undergoes pyrolysis reactions, and some pyrolyzed substances undergo secondary decomposition, volatilizing together with volatile gases and causing changes in sample mass. The third stage is above 650°C, exhibiting a significantly slowed pyrolysis rate, which eventually tends to stabilize. The final weight loss rate within the 1000°C range is less than 50% for all raw materials, indicating that the raw materials exhibit good thermal stability.

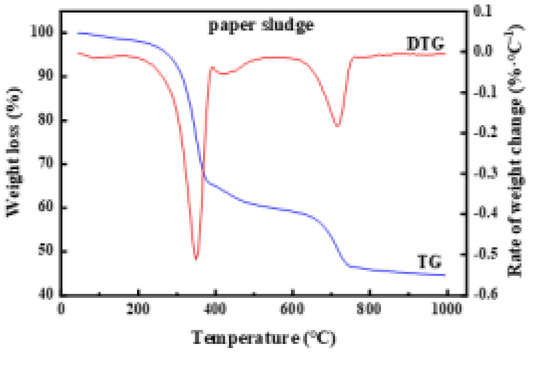


**Figure S1.** TG-DTG diagram of paper sludge.
